# “I Just Get a Different Feeling in This Class”: Belonging as Affective Praxis at a Dutch Urban Secondary Education School

**DOI:** 10.1002/jcop.70073

**Published:** 2025-12-14

**Authors:** Fatma Zehra Çolak, Saro Lozano Parra, Bjorn Wansink

**Affiliations:** ^1^ Department of Education, Faculty of Social Sciences Utrecht University Utrecht Netherlands; ^2^ Department of History and Art, Humanities Utrecht University Utrecht Netherlands

**Keywords:** affect, belonging, pedagogy, racism, student, urban school

## Abstract

While research on the belonging of diverse student populations has grown over the past decades, it tends to overlook the ways subjective feelings of youth are embedded within inequitable societal and institutional structures. Drawing on concepts from critical affect theory, this article studies the experiences of racialized students at an urban secondary school to identify how teachers' affective practices structure their belonging in pedagogical spaces. The study included 16 students (7 female; 9 male), all of whom were adolescents aged between 14 and 17. The findings based on qualitative interviews with students and a constant comparative analytical method illustrate the ongoing role of earlier racialization experiences in figuring into their affective histories and shaping their present encounters with teachers. Student narratives particularly demonstrate how the systematic dismissal of their feelings, presence, and questions by teachers reinforced an atmosphere of alienation in their previous schools. Alternatively, in their current schools, teachers' attunement to students' emotions and diverse learning experiences, along with their ability to tune into students' lifeworlds through humor and friendly banter, seemed to generate an atmosphere of belonging. It is through such humanizing pedagogical practices that students who are marginalized by dominant power structures are affirmed as engaged learners who deserve equitable attention, care, and joy. By studying belonging as an affective praxis, this paper expands our understanding of belonging as a relational and political phenomenon, highlighting the need to develop teachers' critical emotional literacy and praxis.

How come we can't be together and think together in a way that feels good, the way it should feel good?

(Harney and Moten 2013, p.117)

A growing body of research has established belonging as crucial for redressing the harmful consequences of educational inequities for diverse student populations (e.g., Allen et al. 2021). However, being largely normed around notions of whiteness, educational research tends to work from a universalized conception of belonging, centering the experiences of white, often middle‐class students as the norm against which other groups are compared to understand why they are “failing” to fit in (Gray et al. 2018; Kuttner 2023). While acknowledging the role of contextual dynamics in shaping school belonging, dominant psychological approaches to belonging tend to view it as an internal feeling and state, offering little room for exploring subjective feelings of racialized^1^ students as embedded within institutional and societal‐level structures (Ajjawi et al. 2023; Louie et al. 2022).

This paper draws on critical feminist insights on affect (e.g., Ahmed 2004; Wetherell 2012) to move beyond depoliticized notions of belonging and analyze the affective conditions that structure the belonging of racialized youth in everyday pedagogical spaces. An affective lens to belonging allows us to pay attention to the emotional weight of racialization, which shapes the ability of students to feel at ease and engage in transformative learning experiences in school (Çolak et al. 2020; Weiner 2016). More specifically, we focus on the agentic capacity of teacher practices in engineering a learning atmosphere that can either reinforce exclusionary norms or generate humanizing connections attentive to the emotions of racialized students. In this study, we identify teacher practices as affective based on their potential to shape student belonging, and transform classrooms into sites for *otherwise feelings*, those not rooted in alienation and dehumanization (Dernikos et al. 2020). For this, we have interviewed 16 Dutch students of Moroccan and Turkish descent at an urban pre‐vocational secondary education school, exploring how their teachers' affective practices structure their belonging. We study this school as a site of belonging and possibility to defy the dominant pathologization of urban schools as a site of deficit and problems, and demonstrate their potential in addressing issues of injustice (Agirdag and De Leersnyder 2024; Butler et al. 2017; Edwards and Reynolds 2024).

## 1 Critical Approaches to Belonging

Educational and psychological research has extensively explored the concept of *school belonging*, defining it as “the extent to which students feel personally accepted, respected, included, and supported by others in the school social environment” (Goodenow and Grady 1993, p. 80). From this perspective, a *sense of belonging* is seen as a fundamental human need (Baumeister and Leary 1995) and integral to positive student experiences in schools (Allen et al. 2021). However, while highlighting the importance of contextual dynamics and supportive relationships for enhancing a sense of belonging among students, the empirical research on school belonging tends to focus on individual students' ability to fit in and treat their subjective feelings of belonging as internal experiences (Kuttner 2023). As Louie et al. (2022) argue, research on belonging in education, often drawing on Western epistemologies and psychological lenses, measures whether students agree with standard items such as “I feel like a part of my school”, overlooking the inherently relational and political nature of belonging.

It is important to note that traditional psychological approaches to the development of children, such as self‐determination (Ryan and Deci 2000) and attachment theories (Ainsworth et al. 1978), have both acknowledged the role of environmental and interpersonal context in shaping conditions of alienation and belonging. At the same time, scholars drawing on these perspectives are calling for a critical understanding of how wider socio‐political forces, such as racialized hierarchies, affect the experiences of youth marginalized by dominant power structures (López et al. 2022; Stern et al. 2022). Similarly, recent systematic and narrative literature reviews have addressed the role of social, cultural, and environmental factors and barriers such as marginalization and legacies of racism in undermining opportunities to belong among students (Allen et al. 2021; Zaman et al. 2025). As such, a critical and interdisciplinary approach can expand our understanding of belonging as an inherently relational and political phenomenon, one that interrogates how subjective emotions are entangled with broader social and institutional power dynamics (Ajjawi et al. 2023; Gray et al. 2018).

Underlying the importance of institutional and societal structures and conditions for non‐dominant youth to feel a sense of connection, comfort, and belonging, Juang et al. (2018) have emphasized the social group hierarchies which translate into inequitable access to power and resources and sustain mechanisms of racialization. Similarly, Louie et al. (2022) have proposed a *radical belonging framework* to address the limited engagement with structural causes of injustice in existing research on belonging in education. With this, they address the need for developing critical consciousness (i.e., the ability to question injustices) to collaboratively work for justice, centering students' epistemologies and ways of being in learning, and authentic and critical care that receives individuals in their full humanity. In line with social justice‐oriented pedagogies, such as critical pedagogy (Freire 1972), the radical belonging framework entails transformative change and working towards uprooting systems of oppression for building antiracist school communities.

While aligning with the key arguments of these critical frameworks, we do not approach belonging as a fixed, predetermined outcome of equitable structures but as a relational affective praxis situated in humanizing social encounters. Here, we apply the notion of praxis, particularly while referring to belonging‐oriented teacher practices, to highlight their transformative capacity in bending and disrupting patterns of dehumanization (Freire 1972). Approaching belonging as a situated and relational phenomenon, instead of uniform and individual, enables a more nuanced and richer understanding of how students engage and learn under shifting conditions. With this, we seek to create space for multiple stories and imaginative practices that resist universalizing solutions for how to support student belonging (Gravett and Ajjawi 2022).

## 2 Belonging as Affective Praxis

Drawing on an affective approach to belonging, we move beyond the notion of emotions as an internal psychological state, conceptualizing affect as produced in the encounter between bodies within a specific context, rather than being a quality of the body (Alegre Mouslim et al. 2025; Wetherell 2012). Following feminist scholars, we consider affect as encompassing affect, emotion, and feeling in everyday life, and theorize affect as both individually felt and socially and politically situated (Ahmed 2004, 2014a; Van Amsterdam and Eck 2023). We use these concepts interchangeably to avoid reproducing hierarchies between affect as pre‐cognitive bodily intensities and emotions and feelings as subjective experiences, and acknowledge the difficulty of disentangling them in social life (Alegre Mouslim et al. 2025). In studying affective conditions for belonging, we integrate several conceptual ideas: Sara Ahmed (2014a, 2014b) notion of *atmospheres* and Margaret Wetherell's *affective histories* (2012) and *affective practice* (2014). Together, these concepts help analyze how belonging emerges as an affective praxis during social encounters within learning spaces.

Atmospheres refer to “a feeling of what is around”, which underlie their affective and embodied nature (Ahmed 2014b). A learning space, for instance, could feel comfortable and light or tense depending on its material organization as well as social and cultural norms and pedagogical practices that generate different emotional responses (Gravett and Lygo‐Baker 2024). Ahmed (2014b) uses the notion of *atmospheric walls* to explain the inequitable distribution of comfort in predominantly white spaces: “an atmosphere can become a technique, a way for making spaces available for some more than others.” Accordingly, atmospheres serve as an immaterial wall that differentiates between bodies recognized as a legitimate member of a collective and those that “fail” to attune to the mood and become *affect aliens* (Ahmed 2014a, p. 221). Atmospheres have material effects on bodies, meaning they can have behavioral (e.g., silencing, reduced engagement) and affective consequences (discomfort, alienation) for those with less privileged identities (Tan and Faircloth 2023). For instance, institutional and pedagogical practices that exclude nondominant languages can form invisible atmospheric walls, shaping the ability of multilingual students to feel comfortable and engage in a classroom space.

Crafting a classroom atmosphere that enables belonging of diverse student bodies calls for attention to the diverse emotional realities and affective histories that students bring into the learning space. As argued by the feminist psychologist Wetherell (2012), *affective histories* of people may shape the ways they respond to different situations depending on their personal affective biographies and experiences related to their social locations, such as race, gender, language, ethnicity, class and so on. This is particularly crucial while addressing the belonging of racialized youth, given the ongoing social and material impact of race and racism in society and societal institutions (Çolak 2024). For instance, deficit discourses and racializing practices that question the ability of racialized students to learn could seep into classrooms and undermine their belonging through the way they shift the atmosphere (Turcatti 2018). The effect of such discourses and practices can “stick” (Ahmed 2014a) to the bodies of students, shaping the ways they move, navigate, and inhabit learning spaces. However, even though such negative histories may stick in ways that shape the present encounters and future possibilities, they do not necessarily foreclose the potential for alternative realities to emerge (Thiel and Dernikos 2020).

Here, the notion of *affective practice* by Wetherell (2014) is particularly relevant, as it draws attention to the relational and situated nature of affect and emphasizes the agentic potential of actors in the generation of alternative realities. While affective practices are shaped by existing repertoires and past practices, they are not limited to them, allowing potential for alternative connections to emerge even within dehumanizing structures (Wetherell 2014). In classroom contexts, we use affective practice to refer to the attentive relational strategies teachers employ to generate possibilities for students' belonging and engagement. While such affective practices are situated within and shaped by institutional‐ and societal‐level structures and conditions, as well as student responses and histories, we highlight in this study the agentic capacity of teachers in generating alternative atmospheres in classrooms (Lenters 2023).

## 3 Belonging and Teachers' Affective Practices

Existing research on classrooms as affective spaces offers insights into how the constant motion of and encounters between different bodies enable and limit possibilities for diverse student bodies to belong. Studies have, for instance, highlighted the role of policies, institutions, curriculum, and pedagogical practices in shaping the affective atmosphere of learning spaces (Dernikos et al. 2020; Gravett and Lygo‐Baker 2024). While all these actors, including students, co‐create a classroom atmosphere, we particularly focus in this study on the role of teachers' affective practices in crafting it. Previous research has illustrated, for instance, how teachers, through their use of racist jokes, dismissive gestures, and certain tones and looks, can undermine the capacity of students to feel comfortable and engage in a learning space, eventually leading to their disorientation (Çolak et al. 2020; Dernikos et al. 2020; Lozano Parra et al. 2023; Zarabadi 2020).

Even though the affect of teacher practices shift across social, cultural, and institutional contexts, both Dutch and international research highlight the ways non‐normative patterns of behaving and engaging in the classroom (e.g., verbal sharing or excitement during learning activities) are often subjected to a shushing or calling out from a teacher, also referred to as *affective assimilation* (Dernikos 2020; Weiner 2016). To give an example, diverse forms of languaging or responding that are seen as conflicting with dominant classroom norms can reinforce processes of affective assimilation by urging students using heterogeneous linguistic practices (Qin 2018) to act and “feel white” (Dernikos 2020). With this, we do not argue that all classroom management practices reinforce affective assimilation, especially when teachers intentionally adopt responsive practices to enhance the learning experiences of students (Marcucci and Elmesky 2020). Rather, we want to highlight that sanctioning student engagement that does not fit into dominant ways of doing things can form *affective barriers*, holding back student bodies from what they can say and how they can be(long) in learning spaces (Lenters 2023).

Alternatively, teacher practices that indicate attunement to students' diverse affective responses, feelings, and forms of engagement can transform how students listen, engage, and belong (Snaza 2020). Such *attunement* refers to “an alerted sense that something is happening and an attachment to sensing out whatever it is” (Stewart 2010, p.3). Particularly, in the context of student‐teacher encounters, attunement can emerge as a mode of “attention, attending to, being with” (Alegre Mouslim et al. 2025, p. 4). The situated process of becoming attuned is noted by previous research, which indicates how the affective practice of playful teasing can enable conditions for belonging and learning in classrooms (Lozano Parra et al. 2023; Silva and Rogoff 2021) while being considered an act of aggression in another due to its association with harmful stereotypes (Kovats Sánchez et al. 2022). These findings emphasize the role of social and cultural practices (e.g., instructional ribbing), harmful dominant discourses (e.g., deficit perspectives about racialized people) in shaping the emotional responses and engagement of students. As such, a teacher's attunement with their students is likely enabled by their ability to read, sense, and attend to their students' distinct conditions, histories, realities, and emotions related to their social locations (Albuquerque and Pischetola 2024).

Drawing on an ethnographic microanalysis of disciplinary interactions in an urban school in the USA, Marcucci and Elmesky (2020) have identified key moments in which teachers' discursive and affective expressions open up possibilities for student belonging and learning. For instance, a white^2^ teacher in the study, who has a history of positive interactions with his Black student, teasingly uses his student's colloquial expression to facilitate attunement in a moment of perceived misbehavior. With this, the teacher affirms his Black student as an engaged learner rather than relying on societal stereotypes that label Black students as “troublemakers.” Such attunement calls for teacher responsiveness to moment‐to‐moment possibilities as well as the role of affective accumulations that form over time through repeated moments (Lenters 2023; Marcucci and Elmesky 2020). Similar teacher practices are particularly critical in disrupting the harmful effects of deficit‐based narratives on racialized students and in generating humanizing connections and classroom atmospheres.

## 4 The Dutch Educational Context

While educational inequities in the Netherlands continue to affect the schooling experiences of racialized youth, these are often attributed to social and family background or language incompetencies (Sijpenhof 2020; Stevens et al. 2019; Turcatti 2018). Such reasoning perpetuates a deficit‐oriented approach that overlooks unjust features of institutional arrangements, including assimilationist school policies, early‐tracking, the normativity of whiteness, and color‐blind teacher practices (Agirdag and De Leersnyder 2024; Çolak 2024; Hosseini et al. 2021; Weiner 2016). These institutional practices, while deemed neutral, attach an affect of being “less capable” or “deficit” to racialized students from early on, producing the negative affective histories they carry into the classroom (Sieben‐Aduful et al. 2025).

Evidence suggests, for instance, that students with a “non‐Western migration background”^3^ are disproportionately placed in lower educational tracks (e.g., vocational secondary education is attended by only 20% of white Dutch students), while their white peers are more likely to be enrolled in higher tracks (Crul and Schneider 2009; Stevens et al. 2019). These racialized stratification patterns, among other factors, contribute to the high levels of school segregation in large cities (Stevens et al. 2019). Dutch schools with a predominantly non‐white student population, often the lowest educational tracks, have been referred to as “Black” schools in the public discourse since the 1970s (Paulle et al. 2016). The so‐called Black schools are often labeled as problem schools and associated with poorer educational quality, a “problematic” student population, and a teacher shortage due to high dropout rates. In contrast, the so‐called white schools represent the children of middle and higher‐educated white parents and are linked to good quality education and higher educational achievements (Stam 2018).

Recent Dutch and international research findings suggest that dominant discourses of color‐blindness often find their way into the classroom spaces, shaping teacher practices and students' ability to feel at ease due to the invalidation of their ways of doing, being, feeling, and learning (Çolak et al. 2020; Kennedy et al. 2024; Sijpenhof 2020; Weiner 2016). Particularly, in the context of lower‐level secondary schools, attended predominantly by racialized youth, a culture of inequitable disciplining and suspension seems to prevail (Leeman 2007), along with assimilationist practices (Agirdag and De Leersnyder 2024). A deficit association of racialized student bodies with disruptive behaviour, lack of academic interest, and anti‐school norms seems to legitimize such teacher practices (Thijs et al. 2012; Turcatti 2018; van Ewijk 2011; Van Houtte and Van Maele 2012).

At the same time, teacher practices responsive to the emotions and realities of students carry the potential to generate alternative classroom atmospheres (Calabrese Barton et al. 2021; Butler et al. 2017). For instance, recent Dutch research attests to the importance of responsive pedagogical approaches analyzing how teachers in urban secondary schools draw upon them to connect with their students' diverse lifeworlds (Alhanachi et al. 2021; Bonfiglio et al. 2024; Hendriksen et al. 2024; Kennedy et al. 2024). Building on this line of research, the current study centers the subjective feelings and histories of racialized youth to trace how teachers' practices structure conditions of belonging in classrooms. This focus is critical for a student population so often portrayed as “problem youth” in need of discipline, yet whose emotions and experiences are rarely centered (Shaker and Ahmadi 2022; Wekker 2016). In addition, while we highlight racialization as a key mechanism for exploring belonging, students often experience marginalization at the intersections of multiple identities, including culture, gender, class, and religion (Çolak et al. 2020; Turcatti 2018; van den Bogert 2023).

## 5 Methodology

### 5.1 Researcher Positionality and Ethics

This study draws on a critical epistemology, meaning that researchers' lived experiences and social locations play a role in data collection and analysis, as well as the theoretical work. We are a diverse research team in terms of gender, race, ethnicity, country of origin, and home language, and occupy various dominant and non‐dominant positions that have shaped our engagement with issues of belonging, othering, and inequities in education. The first author is a first‐generation Turkish‐Muslim immigrant woman, holding Belgian citizenship and speaking Dutch as a third language. The second author has a bicultural background as a Dutch‐Colombian, which has shaped his interest in studying belonging and the contested dimensions of democracy in citizenship education. The third author identifies as white Dutch and relates to research participants through his past experiences as a student in pre‐vocational secondary education. The second and third authors identify as men, teacher educators and native Dutch speakers. Collaborating across these intersectional positions has afforded us unique angles, shaping our research approach.

While acknowledging the role of our social locations in studying belonging, we are aware of the power we hold as university researchers in our collaborations with schools and youth from non‐dominant communities. Drawing on this awareness, we have prioritized working on building accountable relationships with students and teachers in the school (Smith 2022). The first author, who conducted the interviews, encouraged students to ask questions about both the research process and her academic journey. Students were especially curious about her educational trajectory and seemed to appreciate having space to share their own stories.

This study was conducted following the ethical guidelines of the Ethics Committee of the Faculty of Social and Behavioural Sciences of Utrecht University.^4^ Informed consent was obtained from all participants before any data collection began. For participants under the age of 16, written informed consent was secured from a parent. The process included a detailed explanation of the study's purpose and assurances of confidentiality and anonymity. All participants were assured of their right to withdraw from the study at any time without penalty.

### 5.2 Participants and Procedure

This study draws on in‐depth, semi‐structured interviews with 16 students to explore their experiences and feelings in a pre‐dominantly non‐white pre‐vocational secondary education (VMBO) urban school in the Netherlands. Participants included 7 female (44%) and 9 male (56%) students, of whom 13 (81%) self‐identified as Moroccan and 3 (19%) as Turkish. Although students provided consent to be identified as Moroccan‐Dutch or Turkish‐Dutch in this study, it is important to situate these identifications within a broader racialized context in the Netherlands, where such labels are often externally imposed and associated with being perceived as not fully Dutch (Colak et al. 2022; Moffitt et al. 2025).

By choosing to research the experiences of youth within a single school space, we aimed to provide an in‐depth and nuanced understanding of the micro‐encounters in pedagogical spaces that structure the possibilities for belonging. The vocational track (VMBO) has four levels to which pupils are assigned based on the results of ability tests at the end of primary school called the CITO, and teachers' recommendations. Each VMBO level diploma gives access to a corresponding level in further vocational education. The school offers basic, advanced, and theoretical pathways.

Two teachers, one white Dutch and one Moroccan‐Dutch, who indicated interest in our research, have been especially helpful in facilitating the research by allowing the main researcher to do observations during their teaching hours and introducing the research project to their students. Almost all of the student participants were born and raised in the Netherlands, aged between 14 and 17. A total of seven students followed the theoretical pathway (VMBO‐T), four students followed the basic vocational pathway (VMBO‐B), and five of them followed the advanced vocational pathway (VMBO‐K) (see Table 1 for a participant profile summary). These pathways correspond to a primarily academic track leading to further education (theoretical), a heavily vocational track (basic), and a mixed vocational and academic track (advanced).

**Table 1:** Participant information.

Name^a^	Ethnicity	Gender	Age	Track
Farida	Moroccan‐Dutch	Female	15	VMBO‐T
Nora	Moroccan‐Dutch	Female	15	VMBO‐T
Buchra	Moroccan‐Dutch	Female	17	VMBO‐T
Mariam	Moroccan‐Dutch	Female	16	VMBO‐T
Salma	Moroccan‐Dutch	Female	14	VMBO‐T
Layla	Moroccan‐Dutch	Female	15	VMBO‐T
Yasmin	Moroccan‐Dutch	Female	14	VMBO‐T
Adel	Moroccan‐Dutch	Male	16	VMBO‐KADER
Kerem	Turkish‐Dutch	Male	15	VMBO‐KADER
Said	Moroccan‐Dutch	Male	15	VMBO‐KADER
Yousef	Moroccan‐Dutch	Male	18	VMBO‐KADER
Adam	Moroccan‐Dutch	Male	16	VMBO‐B
Imad	Moroccan‐Dutch	Male	16	VMBO‐KADER
Emre	Turkish‐Dutch	Male	15	VMBO‐BASIS
Taha	Moroccan‐Dutch	Male	16	VMBO‐BASIS
Can	Turkish‐Dutch	Male	16	VMBO‐BASIS

^a^ Pseudonyms used for research participants.

The interviews were conducted by the first author in Dutch, lasting between 30 and 45 min. Interviews were audio‐recorded and subsequently transcribed for analysis. Our primary purpose in conducting these interviews was to gain insights into students' experiences of belonging in their current schools and the role of teacher practices in enabling such experiences in pedagogical spaces. We specifically asked students to narrate specific events and moments with teachers when they felt at ease, comfortable, and seen, to identify the affective practices that generate conditions for belonging in classrooms. We used an open‐ended approach, treating the interview schedule as a guide to allow students to direct the conversations toward topics that mattered to them (Cohen et al. 2007). For instance, we did not initially focus on students' previous experiences with racism in other schools, which took on a much greater emphasis than expected, as the lasting emotional effect of negative past experiences seemed to shape the ways students experience belonging in their current school context. In this sense, our methodological approach is emergent, as we allowed students' lived experiences in schools to guide the direction and focus of the research.

### 5.3 Data Analysis

Following an abductive approach, the data analysis process involved an iterative dialogue between the empirical data and guiding theoretical concepts (Timmermans and Tavory 2012). Using a three‐step approach to data analysis, we first conducted a case‐oriented reading of each transcript to understand each narrative in its entirety (Miles and Huberman 1994). We then produced case summaries to identify recurring subtopics related to our research focus on student belonging and teacher practices. Building on these summaries, we developed an analytic display to support systematic comparison within and across cases, paying attention to counterexamples to strengthen analytic robustness. In the second phase, we employed a constant comparative method, moving between the raw data and emerging interpretations to identify both common and disparate patterns across cases (Charmaz 2014). This stage not only clarified how teacher practices shaped students' feelings, but also generated “unexpected empirical findings,” including the lasting emotional effect of previous experiences with racism and the classroom as a felt space. These insights guided us in refining our analytical lens through the concepts of *affective histories* (Wetherell 2012) and *atmospheres* (Ahmed 2014b), which enabled us to revisit the data and “see things from different angles” (MacLure 2013, p. 174).

The final phase involved a systematic re‐analysis of the entire dataset using this refined theoretical approach. This stage was more deductive, as we used the concepts of *affective histories* and *atmospheres* to perform a focused theoretical interpretation of the data. Through this process, two overarching themes emerged: the production of atmospheres of alienation through racializing practices of dismissal, and the production of atmospheres of belonging through humanizing practices of attunement. While both themes are central, we put greater emphasis on the latter. In line with our framing of the school as a site of possibility, this focus allows for a more detailed exploration of teachers' affective practices that generate conditions for belonging, without overlooking the affective weight of students' past and ongoing experiences of racialization. Overall, our analytical approach reflects an iterative, dialogical, and collaborative process, during which we compared our assumptions, explored alternative interpretations, and checked emerging syntheses against the raw data. These iterative cycles of cross‐checking and reflexive engagement enhanced the trustworthiness of our study (Cornish et al. 2013).

### 5.4 School Climate

The current study focuses on a pre‐vocational secondary education school, anonymized as Future College (FC) in this study, which is situated in an urban neighbourhood in one of the largest cities in the Netherlands. The school attracts a culturally diverse student population from the neighbourhood, often identified as a “problem” region due to an intersection of structural issues, including unemployment and poverty. Despite these barriers, students' accounts suggest that FC offers a generally welcoming atmosphere. Instead of signs promoting surveillance and discipline, the school walls feature affirmative messages, including “all colors are welcome” and quotes from sheroes and heroes selected by the students. The school acknowledges students' religious and cultural identities, for instance, by allowing them to organize communal iftar events during the fasting month of Ramadan. Adam (16) described FC as having a homely atmosphere where everyone is familiar with one another: “It's welcoming, and you get all the information you need about the lesson materials. And it's not a racist school. You don't feel alone here. You're not the only Moroccan at this school.”

For many students, their teachers' responsiveness to their feelings and needs and questions seems to play a key role in their positive impressions of school. Their experiences suggest that many of their teachers show commitment beyond the classroom, including planning home visits and facilitating parents' engagement in school. A key feature of FC is its relatively diverse teaching staff, with racialized teachers comprising roughly one‐third of the faculty. While this appears to have significant implications for students' feelings of comfort at school, many noted that the white teachers at FC were “different” in how they engaged with them when compared with those in their previous schools. As Adel (16) explained, a school‐wide inviting atmosphere seems to prevail, rooted in teachers' ability to connect with them: “You know what I have really noticed? Every teacher is exactly the same… as if they learn from each other… They laugh with you, think with you, are there for you, and want the best for you.”

Despite FC offering a relatively inviting space for students, peer bullying appears to be a key tension in the school, as noted by several students, potentially undermining the ability of some to feel at ease. Nora (15), a member of the student council, explained during the interview that she has witnessed emotional distress among her peers caused by harassment at school. She added that the school management actively intervenes when such incidents are reported. This was echoed by Yasmin (14), who noted, “Yes, yes, there is support, and you can talk to people here if something is bothering you or if you're being bullied or anything like that.” One of the teachers emphasized the complexity of the issue, pointing out that tensions are particularly visible in the hallways between students of different age groups. Highlighting the need for a more systematic approach, the teacher noted that these conflicts can have lasting emotional consequences for some students, ultimately affecting the overall classroom atmosphere.

### 5.5 Findings

Our findings are organized around two interconnected themes that together trace how students' affective histories of racialization “stick” (Ahmed 2004) and continue to shape their educational experiences, while also highlighting how these histories may be reshaped through teachers' affective attunement. First, we examine students' accounts of racialization in their previous schools, highlighting how affective dismissal by teachers contributed to atmospheres of alienation that undermined students' ability to feel seen and heard. We then turn to students' experiences at the FC, where teachers' ability to attune to shifting affective states and diverse learning experiences of students appeared to facilitate humanizing connections, engendering atmospheres of belonging, joy, and care. Together, these themes illustrate how affective histories are often carried into new spaces, while also emphasizing the possibility of transformation through pedagogical practices of attunement.

## 6 Atmospheres of Alienation: Affective Dismissal as Racializing Practice

For many participants, their previous experiences in predominantly‐white, and often upper‐level secondary schools, seemed to bring memories of a sense of alienation and discomfort based on everyday racializing practices. Layla (15) shared how her teacher's dismissal of her questions and selective attention made her feel overlooked and her presence unappreciated:

If you had a question about the work, they would usually say: “Well, you should have been listening,” or “You should have paid better attention.” Okay, then I won't ask any more questions, and because of that, I just thought, I want to get out of here… And also, when it comes to helping, they would first go to someone else and only come to us afterwards, even though we were the first to ask… It's like they just don't care…When I came here, I noticed that it's totally not the case…It felt nice because now if I ask something, I actually get help right away.

Layla's experience suggests that deficit views regarding the academic engagement of racialized students in higher track levels might have shaped her teacher's assumptions about her ability to pay attention to the learning material (Merry and Boterman 2020). In addition, Layla seems to be positioned as an *affect alien* (Ahmed 2014b) within the classroom's racialized atmosphere, where she said, “teachers would really chat with them [white students] and act all friendly and cheerful with them [white students].” Such racializing teacher practices likely served as an affective barrier, silencing and holding Layla's body back from fully engaging in class (Zarabadi 2020).

Students' feelings of being dismissed in their previous schools appeared to materialize in moments when their questions were not taken seriously, or they were asked to wait as their teachers first attended to the questions of another, often more privileged, student. Attributing his teacher's exclusionary approach to the broader deficit views toward students in VMBO, his study track, Adel (16) said:

For them, it's really just explaining it once, done…They really saw the kids from TL [VMBO] as problem kids. You can clearly see it. You say, “I want an explanation,” and then a kid from HAVO [general secondary education] comes in. Then they'll say: ‘You'll have to wait, sorry.’

Adel situated such dismissive teacher practices within what he experienced as an uninviting school atmosphere shaped by rigid rules and norms, with little attention to students' feelings: “Most of the Dutch teachers were usually straightforward about what they wanted. Yeah, just following the rules. They don't really show compassion for the students… It felt more like a prison than a school.” Adel's narrative illustrates how the accumulation of everyday racializing practices, in intersection with exclusionary mechanisms such as tracking, creates an uncaring, carceral pedagogical atmosphere. Yousef (18) experienced a similar sense of alienation at his previous upper‐level secondary school, noting that his teachers were primarily focused on delivering the lesson material and showed little curiosity about students' lifeworlds. He explained that they would leave the class immediately after finishing their teaching:

At my last school, I don't know. It was different. Here, you just have it, you know, a teacher can build a bond with you. At my previous school, they would just teach; they didn't really talk with you. It was just teach, and that's it. They didn't really build a relationship with you.

While the lack of a stronger connection with his teachers seemed to undermine Yousef's engagement in school, Farida (15) shared her feelings of being dismissed and not taken seriously at her previous, predominantly white, upper‐level secondary school. During that time, she said she had attended classes online due to the COVID‐19 pandemic and needed additional guidance while navigating her first year at a new school:

You were not really taken seriously…Yeah, I don't know, people didn't really see me. That was really weird, but people weren't really happy to have me there at the school…They said I wasn't going to pass because I had performance anxiety. My scores weren't super low; they were a little low, but that was also because of Corona, and we had a lot of online classes. I didn't know how to study; it was my first year of high school. I didn't really have the guidance I needed.

While Farida's teachers attributed her “poor performance” to failure‐related stress, her emotional experience of the school atmosphere highlights how her raced and gendered body as a hijab‐wearing Muslim girl was unnoticed when it “mattered,” reflecting a sense of *intersectional invisibility* (Purdie‐Vaughns and Eibach 2008). At the same time, Farida mentioned that she experienced racist stares from peers in the school. Such dehumanizing encounters, rooted in denigrating stereotypes about female Muslim bodies (Gashi and Essanhaji 2023), alongside a lack of guidance and support during a stressful period, likely amplified Farida's feelings of unsafety at her previous school:

It just did not feel safe, like I was in the wrong…When I came to this school, I thought about what they did, and I think that it was really wrong. A school cannot do this, because I also have the right to education. It's not like I'm something completely different. I'm also just a person.

Reflecting on her experiences, Farida shared that the school had used performance anxiety as an “excuse” to remove non‐white students who were perceived as “failing” to fit in. Despite the presence of an alienating school atmosphere that seems to position their bodies as “illegitimate members” and the dismissal of their feelings and experiences, students' experiences indicate how these conditions are often rendered visible, making them leave as if “out of their own will” (Ahmed 2010). While Farida mentioned enjoying the opportunity to continue her education at a school where she said she felt taken seriously, the affective weight of similar earlier experiences can continue to form barriers to how students connect and engage in pedagogical spaces (Turcatti 2018).

## 7 Atmospheres of Belonging: Affective Attunement as Humanizing Practice

### 7.1 Attuning to Shifting Affects

Students' narratives suggest that an atmosphere of belonging and care rooted in humanizing connections prevailed at their current school. Particularly, their accounts highlight teachers' ability to notice, validate, and respond to students' shifting emotional states. Adel (16), for instance, told about how his Dutch language teacher, a white woman, noticed his distress when he became disengaged during her class due to his mother's sickness:

I usually talk a lot and I'm always cheerful, but that day I was just quiet, and she noticed. At the end of the day, she came up to me and asked if I wanted to talk. In that moment, I knew she understood something was wrong and I really needed support. Once I was able to talk, I explained what was going on, and she offered me all the help I needed…It really gave me a good feeling, it's almost like having a friend as a teacher. Teachers here keep an eye on you all the time to see if you're doing okay, and that's nice.

Given Adel's history of inattention in his previous school, such teacher practices carries the potential to repair and humanize his schooling experiences. Adel noted a shared commitment among teachers at the FC to check in with their students and tune into their moods: “I don't really come in with a grumpy look…[Even if] I come in with an angry look, there's always a teacher who makes you happy, because they always start the lesson with a joke.” Adel's recent dream of becoming a teacher attests to the transformative power of his teachers' affective praxis, as he said he wanted to be a teacher to be there for other children the way his teachers were there for him.

In addition to validating and responding to their students' emotions, teachers at the FC seem to actively search for a moment and space to attend to the specific needs and questions of their students. Can (16) shared how his mentors, both racialized women, express their care and attention when they sense his disengagement during a class:

They notice when something is serious and will have a quick chat about it afterwards, or they'll find a moment to talk. They ask about what happened, what caused it, how I'm feeling, and if I need any help…If I'm not feeling well and they see it, they'll ask me during class or right after.

Similar to most of his peers, Can referred to both his white and non‐white teachers at the FC as genuinely engaged with their students and almost all his classrooms as having a pleasant atmosphere, although he mentioned finding it easier to communicate with his Turkish‐speaking teacher, who was well acquainted with his family. While teachers working at the FC seem, in general, attentive to their students' emotions and lifeworlds, it may be easier for racialized teachers to draw on their own affective histories and shared cultural resources in connecting with racialized students (Kennedy et al. 2024).

### 7.2 Attuning to Diverse Learning Experiences

Students' stories indicate that their teachers' attunement to their shifting affective states extends to an awareness and affirmation of diverse learning experiences among students. Adam (16) told, for instance, about a time during Mathematics class when he struggled with an assignment and his teacher, a racialized man, offered one‐on‐one attention to make sure he was on track:

I was really struggling with an assignment in math. And he kept coming back to explain it to me. And I still didn't understand, time after time. Eventually, he said to me: ‘Just stay seated for a bit, and I'll explain it to you in a moment.’ Then I got a one‐on‐one lesson from him at that moment. I understood it right away. Because when you take the time for a student, and really sit down with them, they learn way more quickly than when it is explained to an entire classroom of eighteen, nineteen people…That [gives the feeling] that you're being heard. That they're also giving you attention. No matter who you are…It's really, if you need it, you get it.

Adam's account demonstrates how a teacher's practice of making time to attend to the specific questions of a student likely generates an affect of being validated and worthy of attention as a learner. Such affective practices are particularly powerful for racialized students who are stereotypically associated with learning problems and motivational issues (Çolak et al. 2020). Adam's emphasis here on “no matter who you are” makes such racialized dynamics particularly salient, as it emphasizes how attention, similar to comfort and belonging, is often inequitably distributed in educational settings.

Students noted that teachers' practices of making time often extended beyond regular classroom hours, as teachers not only addressed their individual questions during class but also organized additional tutoring in smaller groups. Buchra (17) explained that while her Economics teacher, a racialized man, made efforts to support her in the large class, such as seating her in the front row for immediate attention, staying on track remained a challenge:

He's just very funny, and he's also really helpful. He actually offers extra tutoring and really pays attention to what I do in class because Economics is definitely not my strongest subject…He tells me all the time, ‘Yeah, we're going to do tutoring.’ We're doing it with a really small group of just girls, four or five girls…A really small group, and I like that a lot…You can pay more attention and focus better, especially since our class is really big.

By reserving additional time and space for their students' diverse learning experiences, these teachers' affective practices counter deficit‐oriented narratives as they affirm their students not only as capable learners but also as humans deserving of equitable attention and care.

### 7.3 Attuning to Affective Atmospheres

Students' accounts demonstrate their attentiveness to different classroom atmospheres, and the ability of teachers to craft a fun and relaxed mood among students through various affective practices, such as the humorous use of students' social and cultural resources and friendly banter. Adam (16) noted that his teachers' ability to integrate humor during lessons created an engaging learning atmosphere:

It's not just looking at a board, and that's what you get. But there's really an atmosphere to it. Yeah, the teacher explains it in a funny way, with funny expressions, and then you laugh along. We really like that kind of atmosphere…They're careful about the kinds of jokes they make. They don't just blurt out whatever comes to mind; they make gentle jokes…But we do have a good laugh in class. And, there are also moments when you need to get serious and work.

Adam's story indicates that teachers, in general, are careful with what kind of humor they employ in crafting an enjoyable atmosphere, while Adel (16) explained how each teacher does it in their own way, not only in terms of how they use humor, but also the moments they choose. For instance, Kerem (15) told about how his teachers first read the general mood in the class before starting with their teaching, emphasizing their ability to resonate with the emotions and lifeworlds of their students:

There needs to be a bit more emotion in the lessons. If they really just keep up with us, then lessons are more fun. Because if it's really just explanations with a low tone, the same regular tone, then we would find it boring…Our teachers look at how the class is, and then they go along with it themselves. For example, with the street language we speak, they also join in making jokes. Like saying ‘daggoe’… And yeah, they kind of keep up with the times as well. They pretty much know what interests us.

Kerem's impressions indicate that teachers enact various relational practices to attune to their lifeworlds outside the school and enhance students' affective experience of learning spaces. What is particularly relevant here is the way that teachers learn informal expressions from their students and draw on them to enact humorous languaging acts, possibly to strengthen their connection with students (Qin 2018).

In general, being able to playfully use their home languages and colloquial expressions with both their white and non‐white teachers seemed to engender feelings of joy and comfort among students. Still, such affective practices carry political implications, especially when enacted by white teachers, whose societal positions of power can transform them into a humanizing response against a political climate that devalues the home languages of these students. Yasmin (14), for instance, talked about how her Technology teacher, a white man, builds an inviting learning atmosphere through affective use of humor, language, and cultural differences:

Yeah, he makes funny jokes and stuff, which makes the lessons a little more fun too. Look, he is [white] Dutch, but he pretends to be a Moroccan…He just talks street language, like kifesh…He would ask things like how it is in Morocco…In the Netherlands, there are a lot of people who hate Turks or Moroccans, just like now, PVV [far‐right Dutch party] has won. If there is a teacher who is Dutch and who wants to kind of acknowledge me, I like that. That he's just fine with me being a Moroccan girl.

For Yasmin, his white teacher's affective and genuine engagement with diverse aspects of her identity likely affirmed her sense of self, countering the dehumanizing anti‐Muslim and anti‐immigrant rhetoric in the Netherlands (Gashi and Essanhaji 2023). In addition, being exposed to affective assimilation at her previous school, where racialized students were punished for speaking in their home languages, Yasmin likely appreciated being allowed to express silenced aspects of her identity in the classroom.

Students' narratives suggest that, in addition to drawing on their social and cultural resources to build a lively atmosphere, their teachers also use various forms of friendly banter, which potentially enables genuine connections, serving as a form of humanizing pedagogy (Qin and Beauchemin 2022). Nora's (15) story shows that her teacher uses affective practices such as playful banter to communicate his high expectations of her and to foster a sense of playful connection between himself and his students:

He can be “annoying” sometimes. He says things like: ‘Yeah, Nora, you should try harder or do more of this.’ Or he'll just tease me a bit. I usually get high grades in his class because I really try my best. One time, I got an 8.5, and I really thought I would get a ten because I did everything perfectly, but then he said, ‘Yeah, you should just try harder, you didn't get a 10.’ He does it on purpose to tease me. He's a fun teacher.

Her teacher's playful affirmation of Nora as a capable and engaged learner likely generated an engaging learning atmosphere., At the same time, such affective practices can serve to counter the pathologizing stereotypes and lower expectations that question the capabilities of racialized and gendered visible Muslim female bodies (Gashi and Essanhaji 2023; Turcatti 2018). Similarly, Salma (14) shared how her teacher, a white man, engages students in learning through diverse affective practices, including humor and friendly teasing, likely encouraging her to engage more freely during his class:

Some teachers are just really grumpy from the very start of the class, and then they get angry, and the students are not in the mood anymore. I don't know, but some teachers look a little dead, like they teach really dry and do not begin the class really happy. This teacher I like begins the class with jokes and stuff, and he is just cheerful during the whole class. I just get a different feeling in this class. It is a bit about how they talk and their body language. I don't know how to explain, he talks in such a way that it feels okay for me as a student to say something…When someone asks a question or something, he also answers in such a way that's not boring. Like when you ask him something, he'll say something like ‘Can't you do it yourself?’ in a funny way, but then he will help you afterwards.

Salma's teacher seemed to activate diverse affective practices, including sarcastic, friendly bantering, to lightly challenge her in figuring out an answer to the question she posed to him before extending his support. In her description of her favorite classroom atmosphere, Salma emphasized that it takes shape based on the emotional state of a teacher at the start of a class, which, as she experienced herself, holds potential in transforming students' feelings and engagement in learning. Such situated affective teacher practices are rooted in the ability of a teacher to attune to the ways students' engagement in the classroom is connected to their feelings (Qin and Beauchemin 2022).

## 8 Discussion

In this study, we have analyzed the experiences of racialized youth across different school settings to explore the role of teacher practices in structuring their belonging. Drawing on conceptual tools from critical affect studies, our findings show that students often encounter racializing teacher practices, suggesting a failure to take students' emotions, questions, and distinct needs seriously. Students' accounts highlight that such dismissive practices made them feel invisible in predominantly white schools, amplifying their sense of exclusion within an already alienating learning atmosphere. Such experiences tend to figure into students' affective histories, shaping how they arrive and engage in pedagogical spaces. At the same time, our research also reveals that the ability of teachers to attune to students' emotions, diverse learning trajectories, and resonate with their students' lifeworlds through humor and friendly banter generates humanizing connections and atmospheres.

While research on belonging often adopts a universal and depoliticized approach, treating emotions as internal features, our study demonstrates first how students' emotional experiences with belonging are entangled with their histories of racialization in their previous schools as well as in society. These findings align with previous research documenting the ways racialized youth are often subject to deficit‐oriented practices and lower teacher expectations (Kennedy et al. 2024; Snaza and Sandlin 2017; Weiner 2016). Students' narratives in our study particularly underscore how these emotional experiences “stick” to their bodies, undermining their capacity to orient themselves and engage in pedagogical spaces (Çolak et al. 2020; Merry and Boterman 2020; Turcatti 2018). We introduce the concept of *affective dismissal* as a form of racializing practice to describe and highlight the stickiness of such dismissal, which often takes the form of inequitable distribution of time, attention, and care during student and teacher encounters. Such racialization experiences are often subtly situated and circulate through pedagogical atmospheres, serving as a technique for alienating racialized students and forcing them to “turn somewhere else” (Ahmed 2006, p. 157). These findings confirm that critical explorations of belonging need to engage more explicitly with historical and ongoing experiences of institutional and systemic injustice and their affective implications on how racialized youth navigate pedagogical spaces (Gray et al. 2018; Juang et al. 2018; Kuttner 2023; Louie et al. 2022).

Secondly, by employing conceptual tools from critical affect theories such as *affective attunement* (Stewart 2010), we illustrate the ways transformative pedagogical practices are rooted in a teacher's ability to sense, align with and tune into subtle affective shifts, forces, differences, possibilities, and atmospheres that enable alternative modes of belonging (Dernikos et al. 2020). We interpret such *affective attunement* as a form of humanizing pedagogical praxis that holds potential for rupturing patterns of racialization through teachers' attention and responsiveness to the feelings, histories, needs, and desires of racialized students for affirmation, care, connection, knowledge, and playfulness (Albuquerque and Pischetola 2024; Dernikos et al. 2023; Hemmings 2005; Lenters 2023). Countering dominant narratives and deficit practices that position these students as academically disengaged “problem” youth, such humanizing pedagogical moves hold potential for repositioning and validating them as deserving of time, attention, and care (Calabrese Barton et al. 2021; Marcucci and Elmesky 2020).

More specifically, students' stories suggest that a teacher's ability to attune to the lifeworlds and feelings of students through affective practices such as humorous languaging acts and playful banter is critical for feeling invited and engaged in a pedagogical atmosphere (Qin 2018). Given their affective histories of being overlooked and invisibilized as “affect aliens”, (Ahmed, 2014), a teacher's commitment to crafting a playful atmosphere likely generates an impression on student bodies that their teachers want them to experience fun in learning. These findings align with research on Black youth in urban education, emphasizing the importance of cultivating possibilities of joy and recognizing the complexity of students' educational experiences to counter their deficit‐framing (Edwards and Reynolds 2024). Additionally, similar authentic pedagogical practices rooted in humor and playfulness likely shift the rigid and hierarchical nature of interactions between a student and a teacher, who display vulnerability and openness by engaging with their students' social and cultural worlds (Qin and Beauchemin 2022).

In particular, the use of humor by teachers drawing on culturally relevant colloquial expressions and friendly banter can affirm students as engaged learners with the capacity to engage in meaningful learning experiences while also validating their multiple selves and resources (Marcucci and Elmesky 2020). Such humanizing pedagogical practices can be particularly transformative and empowering for racialized youth as they recognize both the structural vulnerabilities students are subject to as well as the joy, insights, and resources they bring into classrooms (Qin and Beauchemin 2022). Important to note that, despite its pedagogical potential for fostering caring and humanizing relationships, humorous practices such as playful teasing can also reinforce harmful stereotypes targeting marginalized students (Kovats Sánchez et al. 2022), which attests to its situated and relational nature. Hence, pedagogical humor should be employed with attention, care and intentionality, with teachers staying attuned to the diverse needs, assets, feelings and personhood of their students (Qin and Beauchemin 2022).

### 8.1 Implications for Practice

Our findings call attention to the importance of affect and emotions for rupturing patterns of dehumanization and generating alternative ways of attuning to difference in schools (Zembylas 2014). For this, justice‐oriented pedagogy and practice need to address the complex, varied, and often unpredictable entanglement of emotions and affect with subtle operations of power and structures of racialization that figure into pedagogical encounters (Gravett and Lygo‐Baker 2024). Following Winans (2012), we propose *critical emotional literacy* and *praxis* as valuable pedagogical tools that can nurture the ability of teachers in recognizing the emotions in themselves, their students, and social groups, and to identify their implications. More specifically, an engaged critical inquiry into how emotions inform their relationship to social norms and guide their patterns of attention in everyday school life can provide teachers with opportunities to meaningfully attend and respond to the differences and inequities. For example, Winans (2012) organizes reading and writing sessions for her mostly white pre‐service teachers to support their development of a nuanced vocabulary for a critical analysis of how emotions and affects function and inform their views and beliefs about race and racism. With this, teachers experience a different relationship to their emotions and identities, which can open them to building humanizing connections and relations. Encouraging pre‐service teachers to reflect on the complex entanglement between racialization processes, social and cultural norms, and subjective emotional realities can thus be critical to engendering equitable and humanizing schooling experiences for all students.

### 8.2 Limitations and Recommendations for Future Research

This study has primarily focused on teachers' agentic capacities in shifting and reorienting racialized students' emotional experiences in schools in ways that generate humanizing connections and atmospheres. While centering students' narratives and emotional responses as key to identifying pedagogical practices, we have not fully engaged with students' agentic capacities in, for instance, co‐generating a classroom atmosphere or a meaningful connection with a teacher. Given that belonging is an affective praxis generated within and through relational encounters, future research could use ethnographic approaches to analyze how both teacher and student responses participate in co‐constructing relational dynamics that enable belonging (Qin and Beauchemin 2022). Particularly, an intersectional lens would further illustrate how students' and teachers' affective histories rooted in race, ethnicity, gender, sexuality, and class figure into their encounters and shape pedagogical atmospheres. Our research also highlights how teachers' ability to tune into their students' emotions and learning experiences can enable alternative modes of belonging among racialized students. Yet, we have not explored how teachers can develop such attunement skills. Previous research emphasizes the importance of radical self‐reflection and critical consciousness, arguing that teachers need to have a deep understanding of sociocultural and political contexts that shape their students' experiences (Hosseini et al. 2021). Building on these insights, we call for future studies to more explicitly analyze the intersection between teachers' critical engagement with emotions, critical consciousness, and justice‐oriented pedagogical praxis.

## 9 Conclusion

Despite growing scholarly attention to studying the belonging of diverse student populations, dominant psychological and educational approaches tend to overlook the ways subjective feelings of racialized youth in educational contexts are entangled with institutional and societal structures (Kuttner 2023). In this study, we draw on feminist insights on affect (Ahmed 2014a; Wetherell 2012) to offer a more nuanced and critical understanding of how belonging is enacted as an affective praxis in social encounters within pedagogical spaces. By centering students' embodied experiences of teacher practices across diverse pedagogical atmospheres, we have grounded our analysis in their situated realities and knowledges (Haraway 1988). With this, we have demonstrated how racialized students often arrive at pedagogical spaces carrying personal affective histories shaped by experiences of racialization (Ahmed 2004; Thiel and Dernikos 2020).

Particularly, our findings have illustrated the transformative potential of teachers' affective practices in bending and rupturing patterns of reproduction and dehumanization that shape the orientation, belonging, comfort, and engagement of youth in schools (Ahmed 2014a; Albuquerque and Pischetola 2024; Snaza 2020). These findings offer critical insights into the affective conditions that structure possibilities for belonging during student‐teacher encounters, expanding our understanding of what moves and holds back students. More importantly, they suggest the need for justice‐oriented and anti‐racist practice in education to more seriously engage with affect and emotions as critical forces that enable humanizing connections and atmospheres in everyday school life (Albuquerque and Pischetola 2024; Leibowitz et al. 2010). Ultimately, it is only through turning our attention to the transformative potential of affects and emotions can we truly reimagine justice‐oriented pedagogy and work towards equitable educational futures for every‐body.

## Author Contributions

**Fatma Zehra Çolak:** Writing – review and editing, writing – original draft, methodology, investigation, formal analysis, data curation, conceptualization. **Saro Lozano Parra:** Writing – review and editing, formal analysis. **Bjorn Wansink:** Writing – review and editing, formal analysis.

## Ethics Statement

The study is approved by the Ethics Committee of the Faculty of Social and Behavioural Sciences of Utrecht University. Informed consent was obtained from all the student participants in compliance with university ethics regulations.

## Data Availability

The data that support the findings of this study are available on request from the corresponding author. The data are not publicly available due to privacy or ethical restrictions. The participants of this study did not give written consent for their data to be shared publicly, so, due to the sensitive nature of the research, supporting data is not available.

## Endnotes

1 We use the term “racialized” in this study to highlight the construction of youth with a non‐European background and a non‐white body as Other (El‐Tayeb 2011).

2 In this paper, the term “Black” is capitalized when referring to racial or cultural group identity, reflecting a shared sense of community and history. In contrast, “white” is not capitalized to avoid legitimizing white supremacist ideologies (Laws 2020).

3 This term is widely used in the Dutch context, despite being a racialized label that leaves whiteness and underlying power relations unacknowledged and unexamined (Moffitt et al. 2025).

4 The study is approved by the Ethics Committee, which is based on the documents sent by the researchers as requested by the form of the Ethics Committee and filed under number 23‐0291.
